# Analyzing the efficiency of Chinese primary healthcare institutions using the Malmquist-DEA approach: Evidence from urban and rural areas

**DOI:** 10.3389/fpubh.2023.1073552

**Published:** 2023-02-02

**Authors:** Junxu Zhou, Rong Peng, Yajun Chang, Zijun Liu, Songhui Gao, Chuanjun Zhao, Yixin Li, Qiming Feng, Xianjing Qin

**Affiliations:** ^1^School of Public Policy and Management, Guangxi University, Nanning, China; ^2^Health Policy Research Center, Guangxi Medical University, Nanning, China; ^3^School of Public Policy and Administration, Chongqing University, Chongqing, China

**Keywords:** primary healthcare institutions, relative efficiency, data envelopment analysis, Malmquist index, urban-rural areas, China

## Abstract

**Background:**

China has been increasing the investment in Primary Health Care Institutions (PHCIs) since the launch of the New Health Care System Reform in 2009. It is a crucial concern whether the PHCIs can meet residents' need both in urban and rural with the limited government finance, especially encountering the challenge of the COVID-19. This study aimed to reveal the trend of the primary health service efficiency in the past decade, compare the urban-rural differences, and explore relevant factors.

**Methods:**

DEA and Malmquist models were applied to calculate the health service efficiency of PHCIs among 28 provinces in China, with the input variables including the number of institutions, number of beds, number of health technicians, and the outputs variables including the number of outpatients and emergency visits, number of discharged patients. And the Tobit model was used to analyze the factors on the efficiency in urban and rural. A sensitivity analysis for model validations was also carried out.

**Results:**

The average technical efficiency (TE) of urban PHCIs fluctuated from 63.3% to 67.1%, which was lower than that in rural (75.8–82.2%) from 2009 to 2019. In terms of dynamic efficiency, the urban PHCIs performed better than the rural, and the trends in the total factor productivity change were associated with favorable technology advancement. The population density and dependency ratio were the key factors on TE in both of the urban and rural PHCIs, and these two factors were positively correlated to TE. In terms of TE, it was negatively correlated with the proportion of total health expenditure as a percentage of GDP in urban PHCIs, while in rural it was positively correlated with the urbanization rate and negatively correlated with GDP per capita. Besides, the tests of Mann–Whitney U, and Kruskal–Wallis H indicated the internal validity and robustness of the chosen DEA and Malmquist models.

**Conclusions:**

It needs to reduce the health resource wastes and increase service provision in urban PHCIs. Meanwhile, it is necessary to strengthen medical technology and gaining greater efficiency in rural PHCIs by technology renovation.

## 1. Introduction

The Alma-Ata Declaration, issued by the International Conference on Primary Health Care in 1978, proposed that primary healthcare (PHC) as the basic strategy and key way to achieve the goal of health for all by 2000 (HFA) (1). Primary healthcare institutions (PHCIs) are responsible for the provision of PHC services (2, 3). They make important contributions to the treatment of multimorbidity (4) and chronic diseases (5) as well as to maternal, neonatal, and child health management (6), and are critical to achieving universal health coverage (UHC) and sustainable development (SD) (7, 8).

As one of the countries with the largest number of PHCIs in the world, China has established a primary health care service system covering both urban and rural areas. Especially after the implementation of the New Health Care System Reform policy in 2009, Chinese governments at all levels have invested massively in PHCIs (9, 10). The importance of PHC cannot be overstated since the financial investment in PHCIs in China has reached 215 billion yuan in 2019, according to the China Health Statistical Yearbook 2021. However, the COVID-19 pandemic has highlighted serious weaknesses in health systems in China's health system and a lack of emphasis on PHC, and has demonstrated the need to strengthen primary care capacity (11). PHCIs are not only responsible for basic medical services, but also for epidemiological investigations, sterilization, disinfection, and community checkpoint duty, which puts a lot of pressure on PHCIs and leads to problems such as inefficiency (12). At the same time, Chinese residents' consultation habits are inclined to go to large medical and health institutions, leading to problems such as overloading of large medical institutions and idle resources and insufficient service capacity of PHCIs, which seriously restrict the improvement of the efficiency of PHCIs (13, 14). While the scale of government investment is limited, how to accelerate the development of PHCIs and improve the service efficiency of PHCIs under the established scale of investment is a vital issue to be addressed in the process of deepening the New Health Care System Reform.

Because of their large number, wide distribution, and convenience, PHCIs are accepted and used by a large number of patients and have attracted extensive research by scholars. The service efficiency of PHCIs is one of the most studied elements. In empirical studies, data envelopment analysis (DEA), is widely applied by scholars to analyze the efficiency of such multi-input, multi-output organizations as healthcare institutions (15–18). DEA is a non-parametric method that analyzes the efficiency of PHCIs from the observed data to specify the shape of the production frontier and therefore has no restrictions on the form of the efficient frontier (19). Moreover, DEA can handle multiple inputs and outputs for each evaluated entity in the form of a single efficiency score (20). It follows that DEA has been applied to all different types of hospitals and primary care institutions, etc. (21–23).

Numerous articles and papers used the DEA model for the efficiency measurement of PHCIs for example, Mitropoulos et al. (24) used DEA and bootstrap models to measure the factors that affect the production and economic efficiency of primary health care centers in Greece (24). Zhong et al. (25) evaluated the efficiency and influencing factors of PHCIs in Hunan counties in China, and found that since the implementation of the New Health Care System Reform in 2009, the total health resources of township health centers in Hunan have increased significantly, but most counties have the utilization efficiency of health resources in township health centers was low, and efficiency scores were mainly affected by factors such as population size and urbanization rate (25). Moreover, some scholars have also explored the efficiency of rural PHCIs. Ferrier and Valdmanis used a DEA model to analyze rural hospitals in the United States and concluded that rural hospital efficiency is related to service quality and mix (26). Lin used DEA to retrospectively study micro-efficiency changes in the entire hospital industry in China and found that government input influenced hospital efficiency scores in rural township health centers (27).

It can be seen that there were two problems in the existing literature research on the service efficiency of PHCIs. First, most of the existing studies regarded PHCIs as a whole and studied the service efficiency of the entire PHCIs. Second, the service efficiency of PHCIs in urban or rural was studied separately. And there was no comparative analysis between urban and rural areas, so it was impossible to distinguish the difference in service efficiency of PHCIs in urban and rural areas. But China is a country with an urban-rural dichotomy, and the disparity between the efficiency of urban and rural PHCIs can lead to health inequities. Therefore, based on the research of the above scholars, the objectives of this study are, first, to understand whether there are differences in the service efficiency of primary care institutions in urban and rural areas of China. Second, if there is a difference in efficiency between urban and rural areas, which area is more efficient? Third, to identify what factors influence the efficiency of primary care services in urban and rural areas, respectively. We used the DEA model to quantitatively evaluate and compare the service efficiency of urban PHCIs and rural PHCIs in 28 provinces (including municipalities and autonomous regions) in China, then explored the influencing factors affecting the service efficiency of urban and rural PHCIs through Tobit regression analysis, and finally made comments on the development of urban and rural PHCIs in China.

The rest of this paper is organized as follows. Section 2 is a literature review of the research on the service efficiency of urban and rural PHCIs. Section 3 proposes the DEA-Tobit model and related variables. Section 4 conducts DEA and Malmquist efficiency assessments on the service efficiency of urban and rural PHCIs in China respectively, and analyses the main factors affecting the service efficiency of urban and rural PHCIs. And a sensitivity analysis is applied to verify the correctness of the findings, including Mann–Whitney U test and Kruskal–Wallis H test. Section 5 is summary and conclusion.

## 2. Literature review

The service efficiency of PHCIs is of great significance to the promotion of people's health and the stable development of society and economy, which has always been a concern by scholars (28). DEA model was first used in the healthcare field by Sherman and David (29), and then DEA became the mainstream research method for measuring efficiency in the healthcare field. The research on the service efficiency of PHCIs focuses on the three aspects as follows. First, consider the PHCIs as a whole to conduct service efficiency research, and explore the direction of PHCIs to improve service efficiency. Second, the service efficiency of urban or rural PHCIs has been studied separately. Third, the service efficiency of PHCIs was compared with that of large healthcare institutions, thus identifying the differences in service efficiency between different levels of healthcare institutions.

Many scholars have conducted overall service efficiency studies on PHCIs (24, 25). Zhang and Wang (30) combined DEA and RSR methods to evaluate the service efficiency of PHCIs in 31 provinces in China. It was found that factors such as the technical level and management level of PHCIs were not high enough to restrict the improvement of their service efficiency, and there were regional differences (30). Zhou et al. (31) used TOPSIS and RSR methods to evaluate the level of primary health services and found that the level of primary service provision was uneven in different regions (31). Yan et al. (32) analyzed the efficiency of PHCIs before and after the New Health Care System Reform in China and found that the reform had not achieved the expected goal of promoting the efficiency of PHCIs (32). Some scholars have also separately explored the efficiency of services in urban PHCIs or rural PHCIs (33, 34). Li et al. (35) evaluated the efficiency of township health centers in 31 provinces in China, using the number of township health centers, health personnel, and beds as input indicators, and the number of outpatient consultations, inpatients, and bed utilization as output indicators, and found that the efficiency difference of township health centers is related to the level of economic development (35). Mohammadpour et al. (36) conducted an efficiency analysis of rural primary health care centers in Hamadan, Iran, and found that rural PHCCs in most districts did not reach the highest level of efficiency (36). A few scholars have compared the service efficiency of PHCIs with that of hospitals (37). For example, Osei conducted an efficiency analysis of public hospitals and health centers in Ghana and found that if excess inputs from hospitals were transferred to primary health care facilities, there could be a significant increase in output from primary health care facilities (38). Liu and Zhang (39) used a three-stage DEA model to measure the efficiency of health care services in urban and rural areas of China's health systems, using urban hospitals to represent the urban health care system and primary health care institutions to represent the health care system in rural areas. It was found that compared with rural PHCIs, the imbalance in the allocation of diagnosis and treatment and inpatient services in urban hospitals was more serious (39). Hou et al. (40) compared the efficiency of tertiary hospitals, secondary hospitals, and PHCIs in China and found that the efficiency results showed an “inverted pyramid” (40).

In addition, some scholars have used the DEA-Tobit model to evaluate the efficiency of PHCIs as well as to analyze the influencing factors (41). Marschall and Flessa (42) measured the relative efficiency of rural PHCIs and the factors influencing them in Burkina Faso, Africa, and found that increasing the accessibility of PHCIs would have a significant impact on the efficiency of these institutions. Li et al. (43) analyzed 31 provinces in China through the DEA-Tobit method and found that the service technical efficiency of township health centers is largely affected by the implementation of the New Rural Medical System policy, as well as the illiteracy rate and total dependency ratio of rural residents.

Overall, scholars' research on the service efficiency of PHCIs has never been interrupted, and both theoretical and empirical research has made progress. There is an increasing variety of methods to measure the efficiency of PHCIs, and methods such as DEA, RSR, and TOPSIS have helped to observe the changes in the efficiency of PHCIs in different regions. At the same time, many studies have demonstrated that improving the service efficiency of PHCIs can promote the development of health services, and it is affected by factors such as economic level, dependency ratio, and urbanization rate. Although the research results on the service efficiency of PHCIs are increasingly abundant, there are still problems to be studied: namely, what is the difference between the service efficiency of urban PHCIs and rural PHCIs? What are the factors that affect their efficiency?

In this paper, we used the DEA model to quantitatively evaluate and compare the service efficiency of urban PHCIs and rural PHCIs in 28 provinces in China, then explored the influencing factors affecting the service efficiency of urban and rural PHCIs through Tobit regression analysis, and finally made comments on the development of urban and rural PHCIs in China.

## 3. Methodology

### 3.1. DEA-Malmquist model

DEA is an efficiency evaluation method, which was proposed by the famous operational research scientist A. Charnes (44). This method can be applied to evaluate decision making units (DMUs) with multiple inputs and multiple outputs, and it can effectively evaluate the relative effectiveness of DMUs by analyzing the input and output index data of production decision making units. Different from the parameter method, DEA does not need to presuppose the specific form of the production function. It constructs the frontier production function model through the analysis of the actual observed data, realizes the relative effectiveness evaluation of DMU, and avoids the influence of subjective factors well (45–48).

Efficiency scores can be mathematically described using the DEA approach, as follows (32, 49–51):


(1) $$\begin{array}{l} \max\alpha \\ \{\begin{array}{c} s.\;t.\sum_{j=i}^{n}\lambda_{j}x_{j}+s^{-}=x_{0}\\ \sum_{j=1}^{n}\lambda_{j}y_{j}-s^{+}=\alpha y_{0}\\ \sum_{j=1}^{n}\lambda_{j}=1\\ s^{+}\geq 0,\;s^{-}\geq 0,\;\lambda_{j}\geq 0,\;j=1,\ldots ,\;n \end{array} \end{array}$$

In Equation (1), α is the relative efficiency of the decision making unit (DMU), the larger the value, the more effective the DMU; λ_*j*_ is the proportion of the combination of the DMU that reconstructs an effective DMU combination based on the *j* decision unit; *x*_*j*_ and *y*_*j*_ are the input and output vectors of the λ_*j*_ decision unit, respectively. The *s*^−^and *s*^+^expression input and output slack variables, *x*_0_ and *y*_0_ are the input and output of the DMU respectively. The obtained technical efficiency scores will take a value between 0 and 1, and if TE = 1, it means that DMU is technically efficient.

DEA models can only deal with time series and cross-sectional data, and cannot explain the dynamic changes in the efficiency of decision-making units. The Malmquist index is a frontier analysis that measures the changes in total factor productivity and enables the evaluation of DMUs by years. And the DEA and Malmquist models are used at the same time, which can not only statically observe the efficiency score of a certain year, but also dynamically observe the efficiency change score for consecutive years, ensuring the comprehensiveness of efficiency analysis. The total factor productivity change (TFP) is divided into favorable technology advancement (TECHCH) and technical efficiency change (EFFCH), and further subdivides EFFCH into pure efficiency (PECH) and scale efficiency (SECH) (17, 52).


(2) $$\begin{array}{l} M_{i,\;t+1}\left(x_{i}^{t},y_{i}^{t},\;x_{i}^{t+1},\;y_{i}^{t+1}\right)\\ ={\left[\frac{D_{i}^{t}\left(x_{i}^{t+1},\;y_{i}^{t+1}\right)}{D_{i}^{t}\left(x_{i}^{t},\;y_{i}^{t}\right)}\times \;\frac{D_{i}^{t+1}\left(x_{i}^{t+1},\;y_{i}^{t+1}\right)}{D_{i}^{t+1}\left(x_{i}^{t},\;y_{i}^{t}\right)}\right]}^{\frac{1}{2}} \end{array}$$

In equation (2), $x_{i}^{t},\;x_{i}^{t+1}$ denotes the input vector in period *t* and *t*+1 in region *t* respectively; $y_{i}^{t},\;y_{i}^{t+1}$ denote the output vector in period *t* and *t*+1 in region *t* respectively; $D_{i}^{t}\left(x_{i}^{t},\;y_{i}^{t}\right)$ and $D_{i}^{t}\left(x_{i}^{t+1},\;y_{i}^{t+1}\right)$ denote the distance functions in period *t* and period *t*+1 respectively. If the computed TFP index takes a value >1, then it is thought that TFP is increased from *t* until *t*+1 period; and if it is <1, then TFP is decreased for the same duration.

### 3.2. Tobit Model

To analyze the factors affecting the service efficiency of urban and rural PHCIs, we need to use the technical efficiency scores of urban and rural PHCIs as the dependent variables. The technical efficiency of urban and rural PHCIs ranges from 0 to 1, which has the characteristic of being censored and belongs to the restricted dependent variable. If ordinary least squares are used to estimate parameters, estimates may be biased because the data are not fully represented. The Tobit model is a regression model based on maximum likelihood estimation and is a standard censoring model (53, 54). Which is suitable for the analysis of factors affecting the service efficiency of urban and rural PHCIs.

A Tobit model was established to analyze the influencing factors of the service efficiency of urban and rural PHCIs. In the Tobit model, the dependent variable is the technical efficiency value. The larger the efficiency value, the higher the service efficiency of the PHCIs. Therefore, if the estimated regression coefficient is positive, it indicates that this factor has a positive impact on efficiency. Conversely, if the regression coefficient is negative, it indicates that the factor has a negative impact on efficiency.


(3) $$y_{it}=\{\begin{array}{c} \beta^{T}X_{it}+\varepsilon_{it}>0\\ \beta^{T}X_{it}+\varepsilon_{it}\leq 0 \end{array}$$

In Equation (3), *y*_*it*_ is the technical efficiency value, whose value is the actual observed value when *y*_*it*_> 0; when *y*_*it*_ ≤ 0, the observed value is restricted and takes the value of 0. *X*_*it*_ is the explanatory variable, which takes the actual observed value. β^*T*^ is the vector of parameters to be measured. _*it*_ is the random disturbance term, and ε_*it*_ ~ *N* (0, δ^2^).

### 3.3. Data and variables

#### 3.3.1. Data

The purpose of this study is to measure the service efficiency of urban and rural PHCIs in China since the implementation of the New Health Care System Reform in 2009, so the data from 2009 to 2019 were selected for the study. The urban PHCIs include community health service centers and stations, and rural PHCIs include township health centers and street health centers. There are a total of 34 provinces in China, due to the high urbanization rate in Beijing and Shanghai, the data on township (street) health centers in rural areas were not available in the statistical yearbooks, and these two municipalities were not considered in the efficiency measurement. In addition, considering inconsistencies in data standards, Tibet, Macao, Hong Kong, and Taiwan were excluded from the study. Therefore, urban and rural PHCIs in 28 provinces were finally included. The Input-output data used in this study were extracted from the China Health Statistical Yearbook (2010–2021), and the independent variables were extracted from the China Statistical Yearbook (2010–2020) and the China Education Statistical Yearbook (2010–2020).

#### 3.3.2. Input and output variables for the DEA model

The selection of DEA input-output variables is of great significance to the analysis of health service efficiency. Based on expert consultation and literature reading, three input variables were selected, namely the number of PHCIs, the number of beds, and the number of healthcare technicians, considering the representativeness and accessibility of the selected variables. Among them, the number of institutions and the number of beds represent the physical input of PHCIs, and the number of healthcare technicians represents the human input. Two variables, the number of outpatient and emergency visits and the number of discharged patients in PHCIs, were selected to represent the output of PHCIs, respectively (see Table 1 for details).

**Table 1:** Input and output variables for the DEA- Malmquist model.

**Category**	**Variable**	**Definition**	**Studies**
Input variable	Number of institutions	Number of available community health service centers (stations)/township (street) health centers at the end of the year	e.g., (35)
	Number of beds	Number of available beds at the end of the year	e.g., (25, 35, 40, 41)
	Number of health technicians	Total number of Number of health technical staff at the end of the year	e.g., (25)
Output variable	Number of outpatients and emergency visits	The number of patients coming for outpatient and emergency diagnostic services	e.g., (25, 32, 35)
	Number of discharged patients	The number of discharged patients after hospitalization for various reasons	e.g., (25, 32, 35)

#### 3.3.3. Independent variables for the Tobit model

The service efficiency of healthcare institutions is affected by independent variables such as economy, demographic structure, and urbanization level (32, 55). Based on previous research, this paper mainly selects the following factors as independent variables (see Table 2 for details):

**Table 2:** Variables for the DEA- Tobit model.

	**Variable**	**Obs**	**Mean**	**Std. Dev**.	**Min**	**Max**
**Input variable**						
Urban	Number of institutions	308	1094.58	1142.43	86	6622
	Number of beds	308	6280.44	4642.99	96	22720
	Number of health technicians	308	12835.42	10355.87	468	51100
Rural	Number of institutions	308	1321.15	811.61	143	4745
	Number of beds	308	41348.48	30126.85	2233	135705
	Number of health technicians	308	37999.65	25975.47	1076	113556
**Output variable**						
Urban	Number of visits	308	1.88E+07	2.57E+07	600588	1.36E+08
	Number of Inpatients	308	97325.95	95371.82	126	459021
Rural	Number of visits	308	3.61E+07	2.85E+07	2522889	1.16E+08
	Number of Inpatients	308	1358590	1151690	24301	4950670
**Independent variable**						
Urban & Rural	PGDP (constant 2000 international ¥)	308	42587.82	19636.49	10901.65	118836.50
	URB (people per sq. km of land area)	308	54.23	10.04	29.89	86.50
	POP (people per sq. km of land area)	308	2605.94	1338.55	8.42	5821.00
	SCH (Average years of schooling)	308	8.88	0.72	6.76	12.30
	THEP (%)	252	6.72	1.78	3.16	12.08
	DEP (old and young, % of working-age population)	308	13.75	3.14	7.40	23.80

##### 3.3.3.1. GDP per capita

The degree of economic development of a region tends to influence the increase in government investment in regional PHCIs, which may lead to an increase in service efficiency.

##### 3.3.3.2. Urbanization

Increased urbanization is often accompanied by an increase in the level of education and economic affordability of the local population, which also implies higher quality healthcare services and resources.

##### 3.3.3.3. Population density

The higher the population density, the more pronounced the economic scale effect of government health expenditure, and thus the higher the overall efficiency.

##### 3.3.3.4. Average years of schooling

The higher the level of education of the population, the stricter the regulation of government spending, which in theory will lead to more efficient healthcare spending.

##### 3.3.3.5. Total health expenditure as a percentage of GDP

The proportion of GDP spent on health by local governments affects the scale of investment in health care.

##### 3.3.3.6. Dependency ratio

A larger dependency ratio means that there will be less pressure to supply the labor force and more pressure to supply basic health services, which may have an impact on the efficiency of PHCIs.

## 4. Results and discussion

### 4.1. Results of DEA model

#### 4.1.1. DEA analysis over time

**Figure 1:**
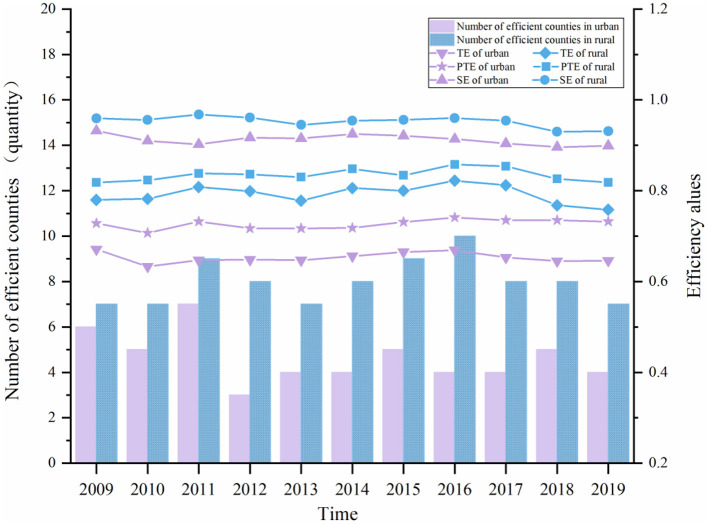
The DEA analysis for PHCIs, 2009–2019.

As shown in Figure 1, in 2009-2019, the mean technical efficiency (TE) of urban PHCIs fluctuated in the range of 0.633-0.671, which was lower than the rural level of 0.758-0.822, indicating that the service efficiency of rural PHCIs was better than that of urban areas. Meanwhile, about 3–7 urban PHCIs operated at optimal levels of scale efficiency (SE) with SE scores of 1.000, and the mean SE ranges from 0.896 to 0.925. And about 7–10 rural PHCIs operating at optimal levels of SE, with the range of average SE from 0.930 to 0.968. And pure technical efficiency (PTE) was low in urban and rural areas.

The analysis results of the efficiency of urban and rural PHCIs showed that the service efficiency of PHCIs in China was relatively low. However, the rural PHCIs have a slightly better TE than urban ones and there are more provinces having a higher SE value in rural PHCIS. It might be because there are more large hospitals in urban areas, which attract many patients. Individuals still tend to visit large hospitals, leading to idle healthcare resources in urban community health service centers (stations), which fail to provide services as efficiently as they should (56). It was also found that the inefficiency of PHCIs in urban and rural areas was mainly due to PTE, indicating that PHCIs were not sufficiently capable of converting input resources into maximum output with the current level of technology, and that input resources were not fully utilized. Therefore, it is necessary to improve the service level and management level of PHCIs, and reasonably formulate their development scale, to improve the efficiency of medical services in various regions.

#### 4.1.2. DEA analysis across provinces

Table 3 shows the DEA scores of urban and rural PHCIs in 2019. For urban PHCIs, the average TE of urban PHCIs in China was 0.646, the average PTE was 0.732, and the average SE was 0.899. Based on the scores, only 4 provinces (14.29%) were efficient, whereas the remaining 24 provinces (85.71%) were inefficient. the TE of the four provinces of Chongqing, Guangdong, Tianjin, and Zhejiang was equal to 1, reaching an efficiency while satisfying PTE and SE. And the return to scale remained unchanged. Among the 24 provinces where DEA was relatively ineffective, 18 provinces had increasing returns to scale, indicating that the community health service centers (stations) in these provinces had a relatively insufficient investment in health care resources. Six provinces had increasing returns to scale, indicating that the medical services of these community health service centers (stations) were not fully utilized under the investment at that time, resulting in a certain degree of waste of resources. Notably, the TE of Inner Mongolia was the lowest among the provinces, and the TE value was merely 0.290. As seen, the reason for the ineffectiveness of its medical service efficiency came more from the ineffectiveness of PTE.

**Table 3:** DEA results of PHCIs in 2019.

**Provinces**	**Urban**				**Rural**			

	**Effch**	**Pech**	**Sech**	**Type of scale inefficiency**	**Effch**	**Pech**	**Sech**	**Type of scale inefficiency**
Anhui	0.695	0.698	0.995	irs	0.877	0.879	0.998	irs
Chongqing	1	1	1	-	1	1	1	-
Fujian	0.719	0.800	0.899	irs	0.711	0.717	0.991	irs
Gansu	0.413	0.439	0.941	irs	0.697	0.724	0.963	irs
Guangdong	1	1	1	-	0.901	0.912	0.988	drs
Guangxi	0.806	1	0.806	irs	1	1	1	-
Guizhou	0.771	0.788	0.979	irs	0.846	0.851	0.995	irs
Hainan	0.483	1	0.483	irs	0.575	0.700	0.821	irs
Hebei	0.479	0.482	0.994	irs	0.631	0.641	0.984	irs
Heilongjiang	0.335	0.354	0.948	irs	0.413	0.430	0.961	irs
Henan	0.629	0.641	0.982	drs	1	1	1	-
Hubei	0.795	0.879	0.905	drs	1	1	1	-
Hunan	0.861	1	0.861	drs	0.944	1	0.944	drs
Inner Mongolia	0.290	0.304	0.953	irs	0.365	0.391	0.933	irs
Jiangsu	0.921	1	0.921	drs	1	1	1	-
Jiangxi	0.533	0.571	0.932	irs	0.880	0.895	0.983	drs
Jilin	0.420	0.561	0.749	irs	0.319	0.342	0.933	irs
Liaoning	0.426	0.430	0.989	irs	0.664	0.715	0.930	irs
Ningxia	0.614	1	0.614	irs	0.661	1	0.661	irs
Qinghai	0.472	1	0.472	irs	0.530	1	0.530	irs
Shaanxi	0.487	0.507	0.959	irs	0.572	0.582	0.982	irs
Shandong	0.563	0.603	0.933	drs	0.746	0.824	0.905	drs
Shanxi	0.332	0.352	0.944	irs	0.475	0.505	0.940	irs
Sichuan	0.921	0.943	0.976	drs	1	1	1	-
Tianjin	1	1	1	-	0.686	1	0.686	irs
Xinjiang	0.458	0.482	0.949	irs	0.894	0.949	0.942	irs
Yunnan	0.656	0.673	0.975	irs	0.848	0.856	0.991	drs
Zhejiang	1	1	1	-	1	1	1	-
Mean	0.646	0.732	0.899		0.758	0.818	0.931	

For rural PHCIs, the average TE of rural PHCIs in China was 0.758, the average PTE was 0.818, and the average SE was 0.931. Based on the scores, 7 provinces (25.00%) were efficient, whereas the remaining 21 provinces (75.00%) were inefficient. Among the 21 provinces where DEA was relatively ineffective, 16 provinces had increased returns to scale, indicating that the townships (street) health centers in these provinces had a relatively insufficient investment in health care resources. Five provinces had increasing returns to scale, indicating that the medical services of these townships (street) health centers were not fully utilized under the investment at that time, resulting in a certain degree of waste of resources. The TE of Jilin was the lowest among the provinces, and the TE value was merely 0.319, and the contributor to the ineffectiveness of its medical service efficiency also came from the ineffectiveness of PTE.

In this analysis, urban PHCIs were less efficient in providing medical services than rural areas. From the perspective of different provinces, the provinces with effective urban PHCIs were Tianjin, Zhejiang, Guangdong, and Chongqing. The majority of these provinces are located in the eastern coastal region of China, which has a more developed economy and a higher level of healthcare services and service capacity, resulting in more efficient services. The provinces with effective rural PHCIs were Jiangsu, Zhejiang, Henan, Hubei, Guangxi, Chongqing, and Sichuan. Among them, Jiangsu and Zhejiang are the eastern coastal provinces of China with more developed economies, which invest more in PHCIs. Henan and Hubei have a higher population density and make fuller use of primary healthcare resources. Guangxi and Chongqing are western provinces in China, which are geographically remote and economically relatively underdeveloped but attach importance to investment in rural PHCIs. Therefore, these provinces have more efficient primary health care services.

### 4.2. Results of Malmquist index

#### 4.2.1. Dynamic change over time

From Table 4, it can be seen that the changes in the Malmquist index and its decomposition for the total urban and rural health care delivery system in the 28 provinces. For urban PHCIs, the average annual growth rate of total factor productivity (TFP) in urban PHCIs from 2009 to 2019 was 1.0%, and there was a trend of fluctuating change, but the overall change was not significant. In addition, as to the root causes of change, there were differences in the trends of different factors of years, with changes in TFP and the efficiency of technological progress showing more similar trends. For rural PHCIs, TFP of healthcare services in rural PHCIs declined by 1.3% during 2009 to 2019. When it comes to the root causes of change, the movement toward TFP of healthcare services in rural PHCIs was similar to the trend of technical efficiency.

**Table 4:** The Malmquist index and its decomposition for PHCIs, 2009-2019.

**Year**	**Urban**					**Rural**				

	**Effch**	**Techch**	**Pech**	**Sech**	**Tfpch**	**Effch**	**Techch**	**Pech**	**Sech**	**Tfpch**
2009–2010	0.931	1.048	0.961	0.969	0.976	1.004	0.959	1.007	0.997	0.962
2010–2011	1.028	1.013	1.035	0.993	1.041	1.028	0.933	1.014	1.014	0.959
2011–2012	1.001	1.065	0.981	1.021	1.066	0.989	1.099	0.998	0.991	1.087
2012–2013	0.998	1.063	0.999	0.999	1.061	0.976	1.026	0.993	0.983	1.001
2013–2014	1.017	0.990	1.003	1.014	1.007	1.035	0.926	1.026	1.009	0.959
2014–2015	1.011	0.982	1.015	0.996	0.993	0.985	1.016	0.982	1.003	1.002
2015–2016	1.016	0.978	1.023	0.992	0.993	1.041	0.944	1.038	1.003	0.983
2016–2017	0.982	1.031	0.997	0.985	1.013	0.993	1.022	1	0.994	1.015
2017–2018	0.982	0.973	0.997	0.985	0.955	0.931	1.047	0.955	0.975	0.975
2018–2019	0.991	1.015	0.987	1.004	1.005	0.983	0.950	0.988	0.995	0.934
Mean	0.995	1.015	1	0.996	1.010	0.996	0.991	1	0.996	0.987

A comparison of the dynamic efficiency of PHCIs in urban and rural areas from 2009 to 2019 illustrated that the dynamic efficiency of urban PHCIs was better than that of rural areas. And they were related to the favorable technology advancement. China has invested heavily in the development of community health service centers (stations) Since the New Health Care System Reform policy, which has promoted their development rapidly. Although the Chinese government has invested heavily in township health centers and village clinics, rural PHCIs are still not attractive to high-quality health personnel. The possible reason is that the economic development level of China's rural areas has been low for a long time, coupled with the cumbersome daily work of rural PHCIs, and the poor salary and working environment (57). Especially in western China, due to the backward economic development, there is a serious drain of medical and health talents in rural areas, resulting in insufficient service capacity and reduced service efficiency of rural PHCIs.

#### 4.2.2. Dynamic change across provinces

As shown in Table 5, there were significant differences in TFP for primary care services among provinces, and overall, urban PHCIs have better TFP values than rural ones. As to urban PHCIs, the TFP changes in most provinces were >1, indicating that the efficiency of healthcare services in urban PHCIs increased universally during the period 2009–2019. As to rural PHCIs, the TFP score for primary health care services was <1 in all but eight provinces, implying a decreasing trend in primary health care services' productivity in most provinces during the period 2009–2019.

**Table 5:** The Malmquist index and its decomposition for PHCIs at the provincial level, 2009-2019.

**Provinces**	**Urban**					**Rural**				

	**Effch**	**Techch**	**Pech**	**Sech**	**Tfpch**	**Effch**	**Techch**	**Pech**	**Sech**	**Tfpch**
Anhui	1.006	1.009	1.007	1	1.016	1.017	0.998	1.015	1.003	1.015
Chongqing	1	1.054	1	1	1.054	1.001	1	1	1	1
Fujian	0.967	0.985	0.978	0.989	0.953	0.978	0.985	0.979	0.999	0.964
Gansu	0.982	1.011	0.982	1	0.993	1.006	0.978	1.010	0.997	0.984
Guangdong	1	1.010	1	1	1.01	0.990	1	0.991	0.999	0.990
Guangxi	1.051	0.997	1.056	0.996	1.048	1	0.982	1	1	0.982
Guizhou	0.974	1.034	0.976	0.998	1.007	0.983	0.947	0.984	0.999	0.932
Hainan	0.984	0.985	1	0.984	0.969	0.975	1.022	0.992	0.983	0.996
Hebei	0.983	1.009	0.983	1	0.991	0.986	0.987	0.984	1.002	0.973
Heilongjiang	1	1.046	1.003	0.997	1.046	0.962	0.956	0.965	0.997	0.920
Henan	1.012	1.013	1.013	0.998	1.025	1.017	1.010	1	1.017	1.028
Hubei	1.011	1.013	1.004	1.007	1.024	1.025	1.024	1.025	1.001	1.050
Hunan	1.040	1.034	1.053	0.988	1.076	1.034	0.976	1.021	1.013	1.009
Inner Mongolia	0.967	1.007	0.971	0.996	0.974	0.970	0.977	0.976	0.994	0.947
Jiangsu	0.993	1.011	1	0.993	1.003	1.005	1.037	1.001	1.004	1.043
Jiangxi	0.992	1.009	0.998	0.994	1.001	0.987	0.956	0.989	0.998	0.944
Jilin	0.981	1.039	1.005	0.976	1.019	0.969	0.989	0.976	0.994	0.959
Liaoning	0.992	1.007	0.992	0.999	0.999	1.022	0.973	1.030	0.993	0.994
Ningxia	1.015	1.015	1	1.015	1.030	0.971	1.015	1	0.971	0.986
Qinghai	0.980	1.010	1.038	0.945	0.990	0.978	0.954	1	0.978	0.933
Shaanxi	1.002	1.016	1.002	1	1.018	1.013	0.972	1.015	0.998	0.986
Shandong	0.992	1.009	0.983	1.008	1.001	0.989	1.027	0.989	0.999	1.015
Shanxi	0.999	1.008	1.002	0.996	1.006	1.019	0.979	1.025	0.994	0.997
Sichuan	0.992	1.047	0.994	0.998	1.039	1.009	0.971	1	1.009	0.980
Tianjin	1.027	1.016	1.024	1.004	1.044	0.964	1.021	1	0.964	0.984
Xinjiang	0.966	1.010	0.967	0.999	0.976	1.041	0.966	1.047	0.994	1.006
Yunnan	0.965	1.010	0.961	1.004	0.975	0.984	0.994	0.985	0.999	0.977
Zhejiang	1	1.016	1	1	1.016	1	1.055	1	1	1.055
Mean	0.995	1.015	1	0.996	1.010	0.996	0.991	1	0.996	0.987

The decomposition of TFP showed that the change in TFP in urban and rural PHCIs was similar to the trend of favorable technology advancement (TECHCH). It can be assumed that TFP is mainly affected by TECHCH, which is consistent with the results of Leng et al. (41). The efficiency of healthcare services in urban PHCIs generally increased, and the increase was mainly due to the improvement of TECH, indicating that urban areas pay attention to the application and improvement of healthcare technology. However, the efficiency of technological progress in rural PHCIs was significantly lower than that in urban areas. It is mainly due to the lack of learning opportunities in township hospitals and village clinics, and the lack of effective supervision of many medical pieces of training, making it difficult to exert practical effects. Moreover, higher-level health institutions have less frequent guidance on the improvement of diagnosis and treatment technology in rural PHCIs. As a result, there has been limited improvement in the treatment techniques and service capacity of rural PHCIs (10). In addition, there was a technological decline in medical and health investments in rural areas. Therefore, in the future, we should focus on improving the internal management level of rural PHCIs, and strengthen the refined management of the medical service system.

#### 4.2.3. Dynamic change across regions

Table 6 shows the variation in TFP of urban and rural PHCIs among different regions. For urban PHCIs, the average TFP of urban PHCIs in the central and western regions was >1, indicating that the average productivity was rising over the 11-years period. The average annual increases were 2.7 and 0.9% respectively, and the main driving force for the increase was technology advancement. TFP in the eastern region was <1, indicating that its average productivity was declining by 0.2%, mainly due to insufficient TE. After further decomposition, it can be seen that it was caused by the lack of PTE and SE. For rural PHCIs, the TFP of rural PHCIs in the eastern region increased, while the TFP of rural PHCIs in the central and western regions decreased. The fastest decline was recorded in the western region, with a decline of 2.7%. The reason for the decline stems not only from a reduction in technology advancement but also from its PTE and SE, which need to be improved.

**Table 6:** The Malmquist index and its decomposition for PHCIs at the regional level, 2009-2019.

**Region**	**Urban**					**Rural**				

	**Effch**	**Techch**	**Pech**	**Sech**	**Tfpch**	**Effch**	**Techch**	**Pech**	**Sech**	**Tfpch**
East	0.993	1.005	0.996	0.997	0.998	1	1.009	1	1	1.009
Central	1.005	1.021	1.011	0.995	1.027	1	0.996	1	1	0.996
West	0.990	1.019	0.995	0.996	1.009	1	0.973	1	1	0.973

The eastern region includes Fujian, Guangdong, Hainan, Hebei, Jiangsu, Liaoning, Shandong, Tianjin, and Zhejiang. The central region includes Anhui, Heilongjiang, Henan, Hubei, Hunan, Jiangxi, Jilin, and Shanxi. The western region includes Chongqing, Guangxi, Guizhou, Gansu, Inner Mongolia, Ningxia, Qinghai, Shaanxi, Sichuan, Xinjiang, and Yunnan.

Through the comparison between urban and rural areas, it can be found that for the eastern region, the dynamic efficiency of rural PHCIs was better than that of urban PHCIs, while for the central and western regions, urban PHCIs were more efficient. The dynamic efficiency of the institutions was better than that of rural PHCIs.

### 4.3. Results of the Tobit model

The Tobit model was used to analyze the factors affecting the DEA efficiency values of urban and rural PHCIs. As shown in Table 7, to reduce heteroscedasticity, we have performed a logarithmic conversion on all independent variables. Population density and dependency ratio were crucial factors affecting the TE of PHCIs in urban and rural areas, and they were both positively correlated. It shows that the greater the population density, the greater the number of patients served by PHCIs, and the greater the scale effect that can be exerted. The higher the dependency ratio, the greater the demand for primary medical and health care, chronic disease treatment and management, and other medical services as the aging population and the minor population increase. The health resources of the PHCIs have been effectively utilized, which has promoted the improvement of the service efficiency of the PHCIs.

**Table 7:** Regression results of the DEA-Tobit panel model.

**Variables**	**TE**		**PTE**		**SE**	

	**Urban**	**Rural**	**Urban**	**Rural**	**Urban**	**Rural**
LnPGDP	−0.0806	−0.182^**^	−0.0212	−0.146^*^	−0.0951^*^	−0.0461
	(−1.263)	(−2.309)	(−0.276)	(−1.679)	(−1.855)	(−1.136)
LnURB	0.257	0.429^*^	0.00594	0.153	0.332^**^	0.108
	(1.257)	(1.789)	(0.0246)	(0.552)	(2.076)	(0.820)
LnPOP	0.0108^**^	0.0112^*^	−0.00195	0.00578	0.0129^***^	0.00768^**^
	(2.109)	(1.746)	(−0.340)	(0.787)	(2.905)	(2.095)
LnEDU	−0.124	−0.109	0.435	0.269	−0.253	−0.00486
	(−0.507)	(−0.363)	(1.331)	(0.654)	(−1.233)	(−0.0295)
LnTHEP	−0.153^**^	−0.0821	0.0617	−0.0512	−0.180^***^	−0.107^***^
	(−2.278)	(−1.054)	(0.695)	(−0.537)	(−3.732)	(−2.989)
LnDEP	0.205^***^	0.138^*^	−0.0257	0.148^*^	0.196^***^	0.0615
	(3.317)	(1.832)	(−0.345)	(1.702)	(3.895)	(1.498)
sigma_u	0.217^***^	0.230^***^	0.338^***^	0.297^***^	0.123^***^	0.0814^***^
	(6.611)	(6.196)	(6.041)	(5.903)	(6.822)	(5.945)
sigma_e	0.0657^***^	0.0795^***^	0.0702^***^	0.0812^***^	0.0571^***^	0.0461^***^
	(19.56)	(17.22)	(16.90)	(14.88)	(19.61)	(17.10)
Constant	−0.262	0.616	0.301	0.720	0.0673	0.530
	(−0.384)	(0.786)	(0.352)	(0.710)	(0.139)	(1.449)
Observations	252	252	252	252	252	252
Number of Provinces	28	28	28	28	28	28

z-statistics in parentheses, ^***^ p < 0.01, ^**^ p < 0.05, ^*^ p < 0.1.

In addition, the TE of urban PHCIs was negatively correlated with the proportion of total health expenditure as a percentage of GDP. The main reason for this is that the Chinese government has increased investment in urban community health service centers (stations) since the New Health Care System Reform. However, due to the rapid growth of resource input and insufficient utilization capacity, health resources have not been used in a timely and effective manner, so the output efficiency has not reached the optimum. Meanwhile, TE in rural PHCIs was negatively correlated with GDP per capita. The possible reason is that rural areas with higher levels of economic development have better transportation conditions, and it is easier to go to large urban hospitals for medical treatment, resulting in idle rural PHCIs (58). And there was a positive correlation between TE in rural PHCIs and the urbanization rate. The possible reason is that the higher the level of urbanization, the more migrant workers. Due to the limitation of geographical distance and economic factors, the left-behind elderly and children mostly stay in rural areas and make full use of the existing health resources in rural areas (59). However, long-term urbanization, low birth rate, and aging trend are prone to the problem of rural hollowing. In the future, some rural PHCIs may face idling, which is not conducive to the stable development of service efficiency of rural primary medical and health institutions.

### 4.4. Sensitivity analysis and model validation

A sensitivity analysis was used to analyze the internal and external validity of the DEA model and Malmquist model results (60). In this study, the output variables of the basic model were removed from the model sequentially (19, 20), and the first and last years were also removed from the model sequentially (61). And the scores of the modified model were used to compare with the original scores to verify whether removing the variables or shortening the study period would lead to significant differences in the efficiency scores.

It is exposed in Table 8, Panel A, that the removal of the number of outpatients and emergency visits and the number of discharged patients from the urban DEA model had a significant effect on the results at the 1% level. And the technical efficiency scores for PHCIs in urban went down from 0.653 to 0.451 and 0.474 respectively. The difference in efficiency scores after removing any of the output indicators is due to the fact that they mix various resource categories. Therefore, significant information removal occurs by excluding each variable. Moreover, we then performed a sensitivity analysis for the reduced study period. The efficiency score went down from 0.653 to 0.651 after removing 2009 data and increased from 0.653 to 0.671 after removing 2019 data, in addition to decreasing to 0.651 after removing 2009 and 2019. The Mann–Whitney U-test showed that by removing 2009 (*p* = 0.891), removing 2019 (*p* = 0.956), and removing 2009 and 2019 (*p* = 0.930), there was no statistically significant change in the distribution of efficiency scores. The Kruskal–Wallis *H*-test showed that there was no statistically significant change in the distribution of efficiency scores between study years (*p* = 0.998).

**Table 8:** Sensitivity analysis of the DEA model.

**Variable/Year removed**	**Average efficiency score**	**Asymp. sig. (Mann–Whitney U)**	**Asymp. sig. (Kruskal–Wallis H)**
**Panel A: Urban**			
None	0.653	-	141.426^***^
Number of outpatients and emergency visits	0.451	−9.901^***^	
Number of discharged patients	0.474	−10.629^***^	
None	0.653	-	0.043
2009	0.651	−0.137	
2019	0.671	−0.055	
2009 & 2019	0.651	−0.088	
**Panel B: Rural**			
None	0.792	-	182.568^***^
Number of outpatients and emergency visits	0.638	−8.038^***^	
Number of discharged patients	0.527	−13.215^***^	
None	0.792	-	0.086
2009	0.793	−0.099	
2019	0.795	−0.165	
2009 & 2019	0.797	−0.285	

*z*-statistics in parentheses, ^***^p < 0.01.

Panel B of Table 8 shows similar results to Panel A. The technical efficiency score went down after removing the Number of outpatients and emergency visits and the Number of discharged patients. And the Mann–Whitney *U*-test and the Kruskal–Wallis *H*-test showed differences in relative efficiency scores after removing any of the output indicators. Sensitivity analysis of the reduced study period showed that the mean efficiency scores increased instead after removing 2009, 2019, and 2009 & 2019 data. The Mann–Whitney *U*-test showed that by removing 2009 (*p* = 0.921), removing 2019 (*p* = 0.869), and removing 2009 & 2019 (*p* = 0.775), the efficiency was no statistically significant change in the distribution of efficiency scores. The Kruskal–Wallis *H*-test showed no statistically significant change in the distribution of efficiency scores between study years (*p* = 0.994).

It is exposed in Table 9, Panel A, that the total factor productivity score for PHCIs in the urban improved from 1.011 to 1.018 and 1.014 after removing the Number of outpatients and emergency visits and the Number of discharged patients. The Mann–Whitney *U*-test and the Kruskal–Wallis *H*-test revealed no statistically significant change in the distribution of total factor productivity scores. We also performed a sensitivity analysis of the reduced study period. After removing data from 2009 and 2019, the Mann–Whitney *U*-test and Kruskal–Wallis *H*-test showed a significant effect on the results at the 5% level, with the total factor productivity score for PHCIs in urban went down from 1.011 to 0.990 and 0.993 respectively, significantly reducing the mode's efficiency score distribution. The Mann–Whitney *U*-test with 2009 and 2019 data removed was not significant. The Kruskal–Wallis *H*-test showed that the total factor productivity scores were statistically different at the 5% level over the study period.

**Table 9:** Sensitivity analysis of the Malmquist model.

**Variable/Year removed**	**Average efficiency score**	**Asymp. sig. (Mann–Whitney U)**	**Asymp. sig. (Kruskal–Wallis H)**
**Panel A: Urban**			
None	1.011	-	0.198
Number of outpatients and emergency visits	1.018	−0.041	
Number of discharged patients	1.014	−0.426	
None	1.011	-	13.102^**^
2009	0.990	−2.180^**^	
2019	0.993	–2.090^**^	
2009 & 2019	1.016	−0.697	
**Panel B: Rural**			
None	0.987	-	19.826^***^
Number of outpatients and emergency visits	1.032	−4.228^***^	
Number of discharged patients	1.007	−2.008^**^	
None	0.987	-	1.447
2009	0.990	−0.377	
2019	0.993	−0.713	
2009 & 2019	0.997	−1.049	

*z*-statistics in parentheses, ^***^p < 0.01, ^**^p < 0.05.

The total factor productivity score of rural PHCIs decreased after removing the Number of outpatients and emergency visits and the Number of discharged patients in Table 9, Panel B. And the Mann–Whitney *U-*test and the Kruskal–Wallis *H*-test showed differences in relative efficiency scores after the removal of any of the output variables. A sensitivity analysis of the reduced study period showed that the efficiency scores of rural PHCIs increased instead after removing data from 2009, 2019, and 2009 & 2019. The Mann–Whitney *U*-test showed that by removing 2009 (*p* = 0.706), removing 2019 (*p* = 0.476), and removing 2009 & 2019 (*p* = 0.294), there was no statistically significant change in the distribution of efficiency scores. The Kruskal–Wallis *H*-test showed no statistically significant change in the distribution of efficiency scores between study years (*p* = 0.695).

## 5. Summary and conclusion

This study applied the DEA-Tobit model to estimate the service efficiency of urban and rural PHCIs in 28 provinces in China from 2009 to 2019. The number of PHCIs, the number of beds, and the number of healthcare technicians were selected as input variables, and the number of outpatient and emergency visits and the number of discharged patients in PHCIs, were selected as output variables of PHCIs and then the factors affecting the service efficiency of urban and rural PHCIs were analyzed. Finally, a sensitivity analysis was used to analyze the internal and external validity of the DEA model and Malmquist model results. The findings are as follows: (1) The service efficiency of China's urban and rural PHCIs was relatively low. There were obvious differences in the efficiency of medical services among regions, and the service efficiency of rural PHCIs was slightly better than that of urban areas. (2) From 2009 to 2019, the service efficiency of urban and rural PHCIs showed a fluctuating trend, and the dynamic efficiency of urban PHCIs was better than that of rural areas. And the change of TFP in urban and rural PHCIs was more related to the changing trend of TECHCH. (3) Tobit regression analysis found that population density and dependency ratio had a positive impact on TE in urban PHCIs, while total health expenditure as a proportion of GDP had a negative impact on TE in urban PHCIs. The urbanization, population density, and dependency ratio had a positive effect on TE in rural PHCIs, while GDP per capita had a negative impact on TE in rural PHCIs.

This is an early study that examines the efficiency of services in both urban and rural PHCIs in the context of China's urban-rural dichotomy. The study's findings suggest that policymakers in China's healthcare sector should focus on improving technical efficiency or management efficiency. In urban areas, there is a need to enhance the rational use of resources, prevent waste, and promote maximum output. In rural areas, there is a greater need to strengthen medical technology and achieve greater efficiency through technological improvements.

This study has some limitations. The inputs and outputs of PHCIs are very complex. Although this study examines the inputs and outputs variable of the primary health care system in two separate parts, urban and rural, it can only partially reflect the service efficiency of PHCIs in both urban and rural areas of China. On the one hand, due to the availability of data, the output indicators only include the quantitative indicators of PHCIs, but not the qualitative indicators of healthcare services, which will be further improved in the subsequent study. On the other hand, this study only analyzed the efficiency of urban and rural PHCIs after the implementation of the New Health Care System Reform policy in 2009 and failed to compare it with the changes in service efficiency of urban and rural PHCIs before the implementation of the New Health Care System Reform policy, which will also be improved in the subsequent study. We will try to obtain more detailed data and conduct a more detailed comparison of the efficiency of urban and rural PHCIs by combining RSR and TOPSIS methods in future research

## Data availability statement

Publicly available datasets were analyzed in this study. This data can be found at: https://data.cnki.net/yearbook/navi?type=type&code=A.

## Ethics statement

This study is entirely an analysis of data from published secondary sources. Since human subjects were not involved, it did not require ethical clearance.

## Author contributions

JZ, RP, and XQ conceived and designed the study. YC and ZL collected data. RP, SG, CZ, and YL analyzed data. RP and QF drafted the paper and other authors provided constructive suggestions and edited the paper. All authors have seen and approved the final version of the paper.
